# The presence of pain in community-dwelling South African manual wheelchair users with spinal cord injury

**DOI:** 10.4102/sajp.v78i1.1600

**Published:** 2022-02-22

**Authors:** Mokgadi K. Mashola, Elzette Korkie, Diphale J. Mothabeng

**Affiliations:** 1 Department of Physiotherapy, Faculty of Health Science, University of the Witwatersrand, Johannesburg, South Africa; 2 Department of Physiotherapy, Faculty of Health Science, University of Pretoria, Pretoria, South Africa

**Keywords:** neuropathic pain, nociceptive pain, spinal cord injury, behaviour of pain, DN4, location of pain

## Abstract

**Background:**

Pain after spinal cord injury (SCI) is common and is likely to continue throughout life with varying levels of severity.

**Objective:**

To determine the presence of pain, the sociodemographic and injury profile of community-dwelling manual wheelchair users.

**Method:**

This quantitative correlational study used a sociodemographic and injury profile sheet and the Douleur Neuropathique 4 Questions (DN4) questionnaire to document demographic, SCI profiles as well as pain characteristics. Pain severity was determined using the Numeric Rating Scale. Data were analysed using the Statistical Package for the Social Sciences (SPSS) v27 at 0.05 level of significance.

**Results:**

The pain rate was 104; 85% of 122 participants and mainly in those with complete SCI (77.9%). Neuropathic pain was more common (76; 62.5%) and significantly associated (*p* < 0.05) with higher pain severity. Pain was mainly in one area of the body (59; 48.4%) but occurring in up to five areas. The most painful area had a mean severity of 6.7/10; was more common in the lower limbs below the injury level (48; 39.4%); and was burning in nature (40; 32.7%).

**Conclusions:**

Pain after SCI is as problematic in the South African context as it is globally. With the rising SCI prevalence in the country, understanding pain and its presentation is important for holistic management of a person with SCI.

**Clinical implications:**

In-depth assessment of pain should be conducted and appropriate management interventions for specific pain types be prescribed to effectively reduce pain.

## Introduction

There is a high prevalence of secondary health conditions (SHCs) in community-dwelling people with spinal cord injury (SCI), with chronic pain being among the leading SHCs (Brinkhof et al. 2016; Piatt et al. 2016). The prevalence of SCI-related pain is estimated to be twice that of the general population (Varghese et al. 2020), with one-third of affected people with SCI (PWSCI) experiencing severe pain (Müller et al. 2016). The actual prevalence rate of SCI-related pain is highly variable, with reported rates between 26% and 96% (Cohen et al. 2018) because of the varying types of pain, as well as other factors such as injury profiles. Recent South African studies report 80% – 94% prevalence rate of pain and further found the multifaceted impact of pain beyond severity and location of pain to include mental health and personal behaviour (Madasa et al. 2020; Pilusa, Myezwa & Potterton 2021a). The experience of pain is unique and specific to each individual because of the multidimensionality of pain, which is emphasised by the dynamic interaction of the biopsychosocial factors of an individual (Todd et al. 2021; Widerström-Noga et al. 2016).

The most common types of pain that PWSCI report are nociceptive and neuropathic pain. Nociceptive pain includes musculoskeletal pain (such as shoulder pain), visceral pain such as abdominal pain and other nociceptive pain such as headaches (Bryce et al. 2012). Musculoskeletal pain is the most common nociceptive pain with 59% of PWSCI reporting this and the pain generally occurs in areas with preserved sensation (i.e. above the level of injury) (Finnerup 2013; Varghese et al. 2020). The behaviour of musculoskeletal pain is usually described as dull, sore, throbbing, tender, aching and cramping (Stanos et al. 2016; Varghese et al. 2020). Visceral pain is only present in a minority of PWSCI within the first 5–10 years following injury and can be difficult to treat (Finnerup 2013). Visceral pain is often atypical because of the sensory fallouts after SCI, resulting in the affected individuals having an altered experience of pain (Varghese et al. 2020).

Neuropathic pain is often chronic and develops within the first year following SCI (Finnerup 2013). This type of pain includes pain at the level and below the level of injury, as well as other neuropathic pain types that are typically unrelated to the SCI itself. It must be noted, however, that these other neuropathic pain types can occur as a consequence of SCI, such as carpal tunnel syndrome with an estimated prevalence rate of 21% – 66% in PWSCI (Varghese et al. 2020). Two-thirds of PWSCI suffer from neuropathic pain, with almost half reporting it as a significant problem (Mashola & Mothabeng 2019). Neuropathic pain can be life-long and typically begins after six months following SCI. It is often described as hot/burning, painfully cold, prickling, numbness, pins and needles, electric shocks, tingling, shooting, squeezing and stabbing (Finnerup 2013; Stanos et al. 2016; Varghese et al. 2020). Most PWSCI have what is coined a ‘mixed pain state’ (Stanos et al. 2016). This type of pain is not yet classified in the International Association for the Study of Pain (IASP) taxonomy but is understood and accepted as a simultaneous overlap of two types of pain symptoms in one individual (Trouvin & Perrot 2019).

Our study is part of a multipronged doctoral thesis (Mashola, Korkie & Mothabeng 2021) which aimed to study pain using the International Classification of Functioning, Disability and Health (ICF) (WHO 2002; Piatt et al. 2016) as its framework because of the dynamic influence of biological, psychological and social factors of SCI-related pain. The ICF places the functioning of a person with disability in a context that involves the interactions between the person’s health, environment, social and personal factors (Mothabeng 2011). The health condition (pain) is affected by the body structures (shoulder biomechanics in the case of musculoskeletal shoulder pain), activities undertaken by PWSCI (such as wheelchair propulsion and transfers), as well as the contextual factors of PWSCI, namely demographic factors such as age and gender, among others (Tran, Dorstyn & Burke 2016). The presence of pain in turn impacts the participation of PWSCI. The interactions between pain as the health condition and the contextual factors are reported in our study.

There is a dearth of literature on pain in South African PWSCI, with few studies highlighting chronic pain as one of the SHCs reported by PWSCI (Mashola, Olorunju & Mothabeng 2019; Madasa et al. 2020; Mashola & Mothabeng 2019; Pilusa et al. 2021a). This literature gap highlights the need to fully understand the presentation of pain after SCI, particularly in a South African context. The aim of our study is to present the presence of pain, with specific objectives being to determine the presence of musculoskeletal and neuropathic pain as well as determine the sociodemographic and injury profile of community-dwelling manual wheelchair users with pain.

## Method

Our study employed a quantitative approach using a cross-sectional quantitative design and took place at participants’ homes or private offices that were within a driving range of 500 km radius from their discharging hospital. This driving range includes Gauteng, North West, Limpopo and parts of the Mpumalanga provinces. These provinces were selected as the distances from the first author’s starting location ranged from 25 km to 100 km within Gauteng and up to 360 km to Mbombela, Mpumalanga. Each province includes suburban, urban, townships and rural areas with residents living in both formal and informal dwellings (Stats SA 2017).

A consecutive sampling method was used where all adult PWSCI were enrolled as they consented to participate in our study. Potential participants needed to be manual wheelchair users with paraplegia discharged for at least six months, irrespective of the presence of pain. People with paraplegia present with partial or full loss of sensory and/or motor function of the trunk and lower limbs (Stevens et al. 2008) and they were targeted as they have full hand function preserved, to be able to manually propel a wheelchair independently. The 6-month post-discharge minimum cut-off period allowed for potential participants to achieve optimum level of independence post-discharge from hospital and for soft tissue changes such as muscle shortening that could possibly occur with prolonged wheelchair use (Ellapen et al. 2017). We excluded consenting participants if they were readmitted to hospital or moved residences beyond the driving range of 500-km radius from their discharging hospital at the time of data collection. Furthermore, participants who did not honour their appointments were also excluded from our study.

The sample size was determined using pain as the instrumental variable to investigate pain and its predictors for the overall doctoral thesis researching the impact of pain on disability and function (Mashola et al. 2021). It was hypothesised that at least 35% of manual wheelchair users experience general pain (Dijkers, Bryce & Zanca 2009), which is a function of no more than eight variables in the doctoral study namely age, gender, type of occupation, years living with SCI, neurological level of injury (NLI), completeness of injury, pectoralis minor muscle length and the presence of scapular dyskinesis. The events per variable (EPV) approach was used to derive the sample size, where EPV > 5, that is, the number of events > 5 × 8 = 40 (Peduzzi et al. 1996). Hence, the sample size equalled 40/0.35 and therefore at least 115 participants. Our study only presents the presence of pain, and the sociodemographic and injury profile of community-dwelling manual wheelchair users and not the predictors of pain.

### Data collection tools

A sociodemographic and injury profile sheet was used to document the participants’ demographic and SCI profile as well as pain characteristics such as the number of painful areas, location of the pain, type and nature of the pain. The painful areas were coded by the most painful to the least painful, namely, first painful area (P1), until the fifth painful area (P5). The severity of pain was determined using the Numeric Rating Scale, a 11-point Likert scale where ‘0’ = no pain and ‘10’ = the most intense pain imaginable that was experienced in the last 24 h. The Numeric Rating Scale has adequate construct and content validity in the SCI population (*r* = 0.38) (Bryce et al. 2007; Dijkers 2010). The 10-item Douleur Neuropathique 4 Questions (DN4) questionnaire was used to classify the type of pain as neuropathic pain or not and has shown high inter-rater reliability values of between 0.70 and 0.96 (Bouhassira et al. 2005).

### Data collection procedure

The databases of the four consenting rehabilitation institutions were perused from May 2018 to December 2018 to identify potential participants. Each potential participant was then contacted via telephone by the first author to invite them to participate in the study in January 2019. Once verbal consent was granted telephonically, the first author travelled to the homes of all the potential participants from February 2019 to March 2020 and an informed consent form was signed on the day of the visit. Although all questionnaires used in our study are self-reported, they were researcher administered to ensure consistency and make sure that the participants understood the questions. There were no language challenges encountered during the administration of the questionnaires.

### Data analysis

Raw data were captured on hard copy capture sheets and transferred to Microsoft Excel during data management. To analyse the data SPSS v27 was used. Descriptive statistics including the mean, standard deviation (SD), data frequencies and percentages were used to report the descriptive statistics. The descriptive statistics include demographic information (such as age and gender), injury information (such as type and cause of injury) as well as pain information (such as location and severity of pain). Fisher’s exact tests and independent *t*-tests were used to report associations and differences between groups, respectively. Testing was done at the 0.05 level of significance and 95% confidence intervals.

### Ethical considerations

This study was conducted in accordance with the ethical principles contained in the current version of the World Medical Association Declaration of Helsinki, and is registered with the South African National Health Research Database (reference GP201806005). Furthermore, our study has been approved by the Faculty of Health Sciences Research Ethics Committee of the University of the Pretoria, South Africa (approval number 125/2018) and permission has been granted by the participating rehabilitation hospitals. Written informed consent has been obtained from all the participants. Confidentiality of both the participants and the participating rehabilitation hospitals is safeguarded by not identifying the participants and giving the rehabilitation hospitals pseudonyms.

## Results

**FIGURE 1:**
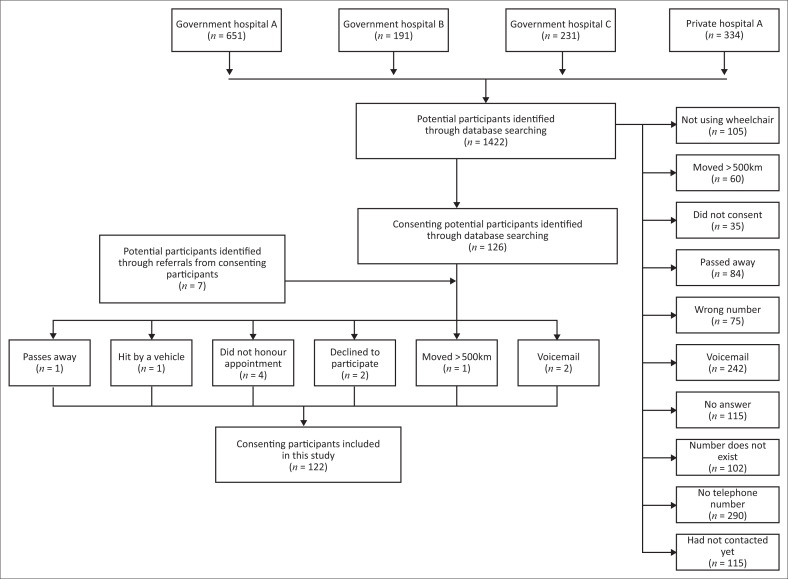
Flow diagram of the included participants in this study.

A total of 122 manual wheelchair users with SCI participated in our study. Figure 1 illustrates how participants were identified and subsequently included.

### Sociodemographic information

The mean age of the participants was 39.7 (SD 11.1) years with the youngest participant being 22 years of age and the oldest 67. The mean age when injured was 32.6 years (SD 10.7), with the youngest age of injury being 14 and the oldest 64. The minimum number of years in which the participants were living with SCI was 11 months and the longest was 40 years, with a mean of 7.1 years (SD 7.1). The participants were predominantly male (*n* = 83, 68.0%) and both genders were injured in their early 30s, with a mean age of 32.7 years (SD = 10.10) for males and 32.4 years (SD = 12.12) for females. Most of our participants resided with their own families (*n* = 62, 50.8%) and in the township area (*n* = 56, 45.9%). Many of the participants were unemployed and relied on government disability grants (*n* = 58, 47.5%). The majority of the participants did not have comorbidities (*n* = 98, 80.3%), and where comorbidities were present, hypertension was the most reported comorbidity (*n* = 6, 4.9%) (Table 1).

**TABLE 1:** Participants’ sociodemographic and injury profile (*n* = 122).

Description	Category	Sub-category	*n*	%
Age (years)	16–30	-	25	20.5
	31–45	-	65	53.3
	46–60	-	26	21.3
	61–75	-	6	4.9
Age when injured (years)	0–15	-	2	1.6
	16–30	-	59	48.4
	31–45	-	44	36.1
	46–60	-	16	13.1
Years living with SCI (years)	< 1	-	3	2.5
	1–5	-	62	50.8
	6–10	-	34	27.9
	11–15	-	9	7.4
	16–20	-	8	6.6
	> 21	-	6	4.9
Gender	Male	-	83	68.0
	Female	-	39	32.0
Residential area	Township	-	56	45.9
	Suburb	-	28	23.0
	Rural	-	26	21.3
	Urban	-	6	4.9
	Plot	-	5	4.1
	Campus	-	1	0.8
Staying with	Own family	-	62	50.8
	Others (*n* = 48, 39.3%)	Extended family	14	11.5
		Friend/partner	3	2.5
		Sibling(s)	12	9.8
		Parent(s)	12	9.8
		Carer	5	4.1
		Roommate/house mate	1	0.8
		Old age home	1	0.8
	Alone	-	12	9.8
Employment status	Unemployed (*n* = 72, 59.0%)	Government disability grant	58	47.5
		Work disability grant	3	2.5
		Dependent on spouse/family	7	5.7
		Assisted by compensation fund lawyer	2	1.6
		Struggling to make ends meet	2	1.6
	Pensioner (*n* = 8, 6.6%)	Government or work pension grant)	5	4.1
		Early retirement pay-out	3	2.5
	Employed (*n* = 32, 26.2%)	Previous work contract not yet ended	1	0.8
		Administration	12	9.8
		Sales	3	2.5
		Finance	2	1.6
		Family business	1	0.8
		Detective (South African Police Service)	4	3.3
		Information technology	5	4.1
		Nurse	2	1.6
		Security	1	0.8
		Manual labour	1	0.8
		Self-employed	9	7.4
	Student	-	1	0.8
Dominant hand	Right	-	116	95.1
	Left	-	6	4.9
Readmission history	No	-	64	52.5
	Yes	-	58	47.5
Reasons for readmission	No readmission	-	64	52.5
	Secondary health conditions (*n* = 55, 45.1%)	Skin	29	23.8
		Urinary	8	6.6
		Respiratory	3	2.5
		Circulatory	2	1.6
		Nervous system (neuropathic pain)	2	1.6
		Skeletal	2	1.6
		Digestive	5	4.1
		Cognitive/mental health	3	2.5
		Reproductive	1	0.8
	Surgical procedures	-	2	1.6
	Further rehabilitation	-	1	0.8
Comorbidities	None	-	98	80.3
	Present (*n* = 24; 19.7%)	Diabetes	2	1.6
		Tuberculosis	2	1.6
		Deep vein thrombosis	1	0.8
		Hypertension	6	4.9
		Hypotension	2	1.6
		Immuno-suppression	3	2.5
		Osteoporosis	1	0.8
		Depression	1	0.8
		Asthma	1	0.8
		Osteomyelitis	1	0.8
		Epilepsy	1	0.8
		Hypercholesterol	1	0.8
		Combination: Hyperthyroidism and Hypercholesterol	1	0.8
		Combination: Thyroidism + Osteoporosis + Arthritis + Lung problem	1	0.8
History of shoulder operations	No	-	117	95.9
	Yes	-	5	4.1
Causes of injury	Traumatic (*n* = 104, 85.2%)	MVA – driver	24	19.7
		MVA – passenger	26	21.3
		Pedestrian vehicle accident	12	9.8
		Motor bike accident	4	3.3
		Gunshot wound	23	18.9
		Stab	2	1.6
		Fall from height	5	4.1
		Object falling on individual	5	4.1
		Agricultural accidents	1	0.8
		Power-paragliding	1	0.8
		Pushed off moving train	1	0.8
	Non-traumatic (*n* = 18, 14.8%)	Tuberculosis spine	9	7.4
		Tumour	5	4.1
		Stenosis	1	0.8
		HIV-related non-traumatic cause	1	0.8
		Fluid accumulation around spinal cord	1	0.8
		SLE-related	1	0.8
Neurological level of injury	T1–T5	-	21	17.2
	T6–T12	-	90	73.8
	L1–L5	-	11	9.0
Completeness of injury	Complete	-	93	76.2
	Incomplete	-	29	23.8

HIV, human immunodeficiency virus; SLE, Systemic lupus erythematosus; MVA, Motor vehicle accident; SCI, spinal cord injury.

### Injury profile

Traumatic causes of SCI were more common than non-traumatic causes (*n* = 104, 85.2% vs. *n* = 18, 14.8%), and this was the case for both males and females (89.2% and 76.9%, respectively). The most common cause of injury was motor vehicle accidents (*n* = 26, 21.3% as a passenger; and *n* = 24, 19.7% as a driver) and gunshot wounds (*n* = 23, 18.9%). The majority of the participants had complete injuries (*n* = 93, 76.2%), with neurological levels of injury between T6 and T12 (*n* = 90, 73.8%) and they were right hand dominant (*n* = 116, 95.1%). Most of the participants had no previous history of readmission back to hospital (51.5% vs. 47.5%), and those who did have a readmission history were mostly readmitted for skin secondary health complications (*n* = 29, 23.8%) (Table 1).

### Pain presentation

**FIGURE 2:**
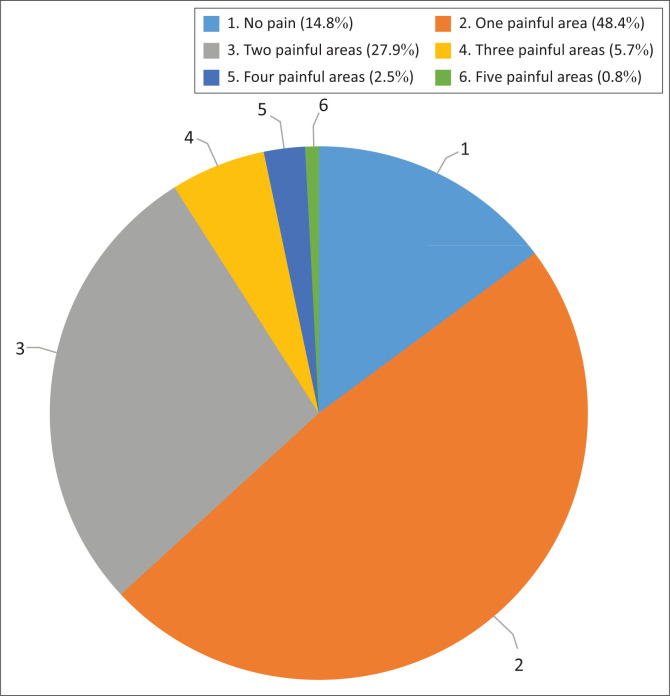
Number of painful areas.

We found that 85% of our participants experienced pain at the time of our study (*n* = 104), with the majority of the participants experiencing neuropathic pain more than musculoskeletal pain (*n* = 65, 62.5% vs. *n* = 39, 37.5%). Most of the participants reported the pain to be in one area (*n* = 59, 48.4%) and up to five painful areas as depicted in Figure 2. Pain was mainly reported by the participants between the ages of 31 and 45 years (*n* = 53, 51.0%) and living with SCI between one and five years (*n* = 56, 53.9%). The presence of pain was mainly in the T6–T12 NLI (*n* = 78, 75.0%) and in participants with complete injuries (*n* = 81, 77.9%).

#### Type and location of pain

The 104 participants who reported pain were asked to locate all their painful areas, describe the pain and rate the pain severity. Neuropathic pain was more common in the most painful area (P1: *n* = 76, 73.1%) and second most painful area (P2: *n* = 24, 23.1%), while the third and fourth painful areas (P3 and P4) were more nociceptive than neuropathic pain (*n* = 7, 6.7% and *n* = 3, 2.9%, respectively). Only one participant (0.96%) reported P5 as a muscular ache in the hands. Although pain occurred throughout the whole body, the lower limbs below the NLI was the most common location of pain in P1 and P2 (39.4% and 10.6% respectively), while two participants reported the non-dominant shoulder and the upper back above the NLI for P3 and P4, respectively (1.9%, respectively) (Table 2). We found that P2 was significantly associated with neuropathic pain below the level of injury, where 10 of the 11 participants (90.9%) who reported this location had complete injuries (Fisher’s exact = 20.36, *p* = 0.041).

**TABLE 2:** Type and location of pain (*n* = 104).

Type of pain	Sub-type of pain	Pain location	P1		P2		P3		P4	
			*n*	%	*n*	%	*n*	%	*n*	%
No pain	-	-	0	0	59	56.7	93	89.4	100	96.2
Nociceptive pain	Musculo-skeletal pain	Shoulder (dominant)	1	1	1	1	1	1	0	0
		Shoulder (non-dominant)	1	1	5	4.8	2	1.9	1	1
		Shoulder (bilateral)	0	0	5	4.8	1	1	0	0
		Elbow(s)	1	1	1	1	0	0	0	0
		Wrist(s)	1	1	2	1.9	0	0	0	0
		Hand(s)	1	1	0	0	0	0	0	0
		Upper back (above NLI)	10	9.6	3	2.9	1	1	2	1.9
		Lower back (above NLI)	2	1.9	1	1	0	0	0	0
		**Sub-total**	**17**	**16.5**	**18**	**17.4**	**5**	**4.9**	**3**	**2.9**
	Visceral pain	Thorax (Chest)	5	4.8	3	2.9	1	1	0	0
		Abdomen	3	2.9	0	0	1	1	0	0
		**Sub-total**	**8**	**7.7**	**3**	**2.9**	**2**	**2**	**0**	**0**
	Other	General body pain	1	1	0	0	0	0	0	0
		Groin pain	1	1	0	0	0	0	0	0
		Headache	1	1	0	0	0	0	0	0
		**Sub-total**	**3**	**3**	**0**	**0**	**0**	**0**	**0**	**0**
**Total**	**-**	**-**	**28**	**26.9**	**21**	**20.2**	**7**	**6.7**	**3**	**2.9**
Neuropathic pain	At-level	Back (injury site)	11	10.6	3	2.9	1	1	0	0
		**Sub-total**	**11**	**10.6**	**3**	**2.9**	**1**	**1**	**0**	**0**
	Below level	Lower back (below NLI)	11	10.6	6	5.8	0	0	0	0
		Waist (below NLI)	3	2.9	1	1	1	1	0	0
		Hip(s)	6	5.8	2	1.9	0	0	0	0
		Buttock area(s)	4	3.8	1	1	1	1	1	1
		Lower limbs (below NLI)	41	39.4	11*	10.6	1	1	0	0
		**Sub-total**	**65**	**62.5**	**21**	**20.3**	**3**	**3**	**1**	**1**
**Total**	**-**	**-**	**76**	**73.1**	**24**	**23.1**	**4**	**3.9**	**1**	**1**

NLI, neurological level of injury.

* , Significant association with complete injuries (*p* = 0.041).

#### Behaviour of pain

The most common nature of the pain was burning in P1 (32.7%), while joint aches were the most common pain in P2 (11.5%) and P3 (4.8%). Two participants (1.9%) reported muscular aches as the common nature of pain in P4. The one participant who reported P5 described it as a muscular ache. One participant could not describe the 6 out of 10 abdominal pain they experienced. We found discrepancies with the way participants allocated the terms ‘cramping’ and ‘stabbing’ as described in the footnote below (Table 3). The total results are highlighted in Table 3. One participant described their at-level neuropathic pain as ‘cramping’ for P1, which is an accepted description of muscle cramps in nociceptive pain. Five participants described the pain in their wrist, shoulders, lower back (above level of injury), thorax and upper back (above level of injury) as ‘stabbing’– which is an accepted description of neuropathic pain and not nociceptive pain. In anticipation for this discrepancy, the DN4 was used to determine the overall presence of neuropathic pain and the results are shown in Table 4. Similar to P1 in Table 3, the majority of participants reported neuropathic pain (*n* = 65, 62.5%) as compared to nociceptive pain (*n* = 39, 37.5%) and mainly described the nature of the pain as burning (*n* = 61, 93.8%).

**TABLE 3:** Behaviour of pain (*n* = 104).

Pain behaviour	P1		P2		P3		P4	
	*n*	%	*n*	%	*n*	%	*n*	%
**No pain**								
**Nociceptive pain**	0	0	59	56.7	93	89.4	100	96.2
Pulling	2	1.9	1	1	0	0	0	0
Thumping	1	1	0	0	0	0	0	0
Cramping	3	2.9†	0	0	1	1	0	0
Deep pressure	6	5.8	1	1	0	0	0	0
Dull	5	4.8	0	0	0	0	0	0
Ache: Muscular	6	5.8	1	1	1	1	2	1.9
Ache: Joint	4	3.8	12	11.5	5	4.8	1	1
Throbbing	1	1	0	0	0	0	0	0
Indescribable	1	1	1	1	0	0	0	0
**Total**	**29**	**27.9**	**16**	**15.5**	**7**	**6.7**	**3**	**2.9**
**Neuropathic pain**								
Burning	34	32.7	11	10.6	3	2.9	1	1
Painful cold	1	1	1	1	0	0	0	0
Electric shocks	5	4.8	1	1	0	0	0	0
Tingling	0	0	1	1	0	0	0	0
Pins & needles	13	12.5	5	4.8	0	0	0	0
Stabbing	20	19.2	9	8.7‡	1	1	0	0
Stinging	1	1	0	0	0	0	0	0
Itchy	1	1	1	1	0	0	0	0
**Total**	**75**	**72.1**	**29**	**27.9**	**4**	**3.9**	**1**	**1**

† , One participant described their back pain at-level of injury, as cramping;

‡ , Five participants described their pain above the level of injury as stabbing.

Highlighted areas depict the discrepancies in the terminology used by the participant

**TABLE 4:** Douleur neuropathique 4 descriptive statistics (*n* = 65).

Description	*n*	%
Burning	61	93.8
Painful cold	17	26.2
Electric shocks	46	70.8
Tingling	31	47.7
Pins and needles	54	83.1
Numbness	22	33.8
Itching	24	36.9
Hypoaesthesia to touch	62	95.4
Hypoaesthesia to prick	64	98.5
Worsened by brushing	12	18.5

#### Severity of pain

The mean pain severity for P1 was 6.7 (SD = 2.3), 2.4 for P2 (SD = 3.0), 0.6 for P3 (SD = 1.7) and 0.2 for P4 (SD = 0.9). The one participant who reported P5 in the hands rated the severity of the pain as 3.5 out of 10. We found significant differences in P1, P2 and P3 severity among participants who reported the presence of neuropathic pain and those who did not. Neuropathic pain as per the DN4 (Table 4) showed significantly higher pain severity means than musculoskeletal pain (*p* < 0.001 for P1, P2 and P3, respectively) (Table 5). There was a mean difference of 2.2 points for P1, 2.5 points for P2 and 0.88 points for P3. P1 severity also showed significant differences between male and female participants with females reporting a higher overall pain severity by 0.98 points (*p* < 0.05). We also found significant differences in P2 severity between participants with complete and incomplete SCI. Those with complete SCI reported significantly higher pain severity by 1.27 points than those with incomplete SCI (*p* < 0.05) as shown in Table 6. We did not find any other associations between the pain information and demographic nor injury profile.

**TABLE 5:** Differences between musculoskeletal and neuropathic pain (*n* = 104).

Pain	Independent *t*-test statistic	*p*	Group	*n*	Mean	Std. dev.
P1 severity	−5.468	0.000	Musculoskeletal pain	39	5.35	2.16
			Neuropathic pain	65	7.55	1.87
P2 severity	−5.115	0.000	Musculoskeletal pain	39	0.80	1.74
			Neuropathic pain	65	3.30	3.25
P3 severity	−3.465	0.001	Musculoskeletal pain	39	0.00	0.00
			Neuropathic pain	65	0.88	2.04

Std. dev., standard deviation.

**TABLE 6:** Differences between gender and completeness of injury with regard to pain severity (*n* = 104).

Pain	Independent *t*-test statistic	*p*	Group	*n*	Mean	Std. dev.
P1 severity	−2.116	0.037	Male	71	6.41	2.31
			Female	33	7.39	1.98
P2 severity	2.124	0.039	Complete SCI	81	2.64	3.15
			Incomplete SCI	23	1.37	2.33

SCI, spinal cord injury; Std. dev., standard deviation.

## Discussion

Pain is a major problem after SCI and the high presence of pain that we found in our study is similar to that found in the literature (Adriaansen et al. 2013; Hassanijirdehi et al. 2015; Madasa et al. 2020). Our findings of high rate of pain are similar to those reported by Madasa et al. (2020) who found that 80% of their participants in Cape Town reported the presence of pain after SCI. Our findings are consistent with global literature in that neuropathic pain is the most common type of pain; in the lower limbs and ‘burning’ is one of the major symptoms (Adriansenn et al. 2013; Gustin et al. 2014; Ullrich et al. 2008; Varghese et al. 2020). Although the lower limbs were the most common location of pain in our study, we would like to emphasise that pain is common throughout the body. By broadening the identification of pain from most severe to least severe, it may assist in addressing all the pain complaints reported by PWSCI. As in other studies, pain was present in more than one area of the body (Müller et al. 2016; Richardson et al. 2021) as well as having both neuropathic and nociceptive pain occuring simultaneously, as supported by the concept of mixed pain (Trouvin & Perrot 2019). We also found that SCI occurs predominantly in males (Brinkhof et al. 2016; Gabbe & Nunn 2016) and similar to Day and Thorn (2010), women reported significantly higher severity of pain than men. Study findings on gender and SCI-related pain are inconsistent. However, general research in females demonstrate the anti-dopaminergic effect of oestrogen, which suggest females as being more inclined to report pain and the higher prevalence of mood disorders as contributors to a higher pain prevalence than men (Müller et al. 2016).

Similar to other studies, SCI was common in the early middle-aged population (Brinkhof et al. 2016; Madasa et al. 2020; Pefile, Mothabeng & Naidoo 2018). The average age of people living with SCI is like local findings (Joseph & Nilsson Wikmar 2016) and is much younger compared to international findings (Gross-Hemmi et al. 2021). We must note that we did not find a significant association between age and the presence of pain, like findings by Hassanijirdehi et al. (2015). Nevertheless, middle age is the economically viable age, and our participants could be participating in the labour market had it not been for the SCI. We found a very high rate of unemployment similar to the findings by Mann et al. (2013), and although it is disconcerting, it is not surprising as SCI is known to leave the majority of affected individuals without employment (Pefile et al. 2018). The opportunity for gainful employment after SCI is limited, even without the added burden of SHCs such as pain, which according to Mann et al. (2013), increases not only the ‘humanistic’ burden, but the economic burden as well.

Pain is identified as a significant barrier to an individual’s potential to earn a living in terms of employment and being financially independent (Tran et al. 2016). As in Pefile et al. (2018) who conducted their study in Kwa Zulu Natal, we also found the majority of participants resided in townships (peri-urban) areas. However, unlike an American study by Day and Thorn (2010), we did not find any association between the presence of pain and residing in a rural area as well as being unemployed. Of course, we must note that rural Alabama in USA where the study was conducted is a different context to rural areas in South Africa, which has a marked socio-economic disparity (Pilusa, Myezwa & Potterton 2021b). We agree that there are general healthcare issues within rural populations and that the burden of living with pain may be worsened by numerous barriers such as access difficulties and limited medical services. However, more research is needed to confirm this association in a South African context.

The majority of our participants had been living with SCI between 1 and 5 years, as in the findings by Brinkhof et al. (2016). This finding, however, did not yield a significant association. It has been suggested, however, that the severity of pain increases as the duration of years living with SCI increases (Adrianssen et al. 2013). Pain is a phenomenon that is most likely than not, ongoing and has the potential to be chronic when left untreated. Pain, when left untreated has the potential to worsen in nature, thus increasing in severity. Mann et al. (2013) further reports that the health status and QOL of a person with SCI experiencing pain decreases significantly as the pain severity increases. This is concerning as it suggests that the longer individuals live with SCI, the worse the pain will be and the worse their overall health status will become. Pain management interventions should therefore be prioritised to manage SCI-related pain, especially because Middleton et al. (2012) reported improvement in life expectancy after SCI as compared to 10 years ago. It would be a more meaningful life without the burden of increasing pain.

Pilusa et al. (2021a) have highlighted the presence of comorbidities in PWSCI, and we have conversely found that our sample had a low presence of comorbidities and other health problems. Perhaps it is because the focus of our study is the presence of pain and we asked participants to report health problems other than pain, which is almost always reported in both local and international literature as a current health problem (Joseph & Nilsson Wikmar 2016; Madasa et al. 2020; Mashola & Mothabeng 2019; Gabbe & Nunn 2016). With the low rate of health problems in our study, it was therefore unsurprising that we found that the majority of our participants were never readmitted back to hospital after their initial discharge. Gabbe and Nunn (2016) also found that only 39% of their participants were readmitted in the first 2 years after injury, and although lower, we had previously found an 18% readmission rate during a 4-year period (Mashola et al. 2019) as a result of SHCs. Of course, a low readmission rate does not lessen the severity of SHCs and their impact on PWSCI. However, the finding is useful as it further supports SHCs as the cause of readmission in PWSCI (Joseph & Nilsson Wikmar 2016; De Jong et al. 2013).

Similar to the findings by Brinkhof et al. (2016), SCI in our study was mostly because of traumatic causes (mainly motor vehicle accidents). Earlier South African studies have reported both traumatic (Joseph & Nilsson Wikmar 2016) and non-traumatic (Pefile et al. 2018) injuries. In our study, we also found that the majority of the participants had complete injuries, similar to Joseph and Nilsson Wikmar (2016), whereas Pefile et al. (2018) found incomplete injuries to be in the majority. Although both were South African studies, Kwa-Zulu Natal, where the study by Pefile Mothabeng and Naidoo was based, has an increased retroviral HIV infection rate, thus leading to the majority of their participants having non-traumatic and incomplete injuries. We found pain to be more prominent in participants with complete injuries than incomplete, as in the findings by Brinkhof et al. (2016). We found that this relationship did not yield significant findings with the most painful area of pain (P1) but was significant with the second most painful area (P2). It must be noted that our population predominantly consisted of people with complete SCI and the comparisons between the reporting of pain severity by people with complete and incomplete SCI must be considered carefully. Ullrich et al. (2008) did not find a significant relationship between pain and completeness of injury as well and explained that psychological factors (such as mood) are better predictors of pain than SCI characteristics (Ullrich et al. 2008).

We cannot adequately report on the level of injury being a predictor of pain as our study only focused on people with paraplegia. Nevertheless, Hassanijirdehi et al. (2015) also did not find any association between level of injury and the presence of pain, despite the inclusion of people with tetraplegia. This finding may suggest that pain is a common problem among PWSCI, regardless of the level of injury.

### Strengths and limitations

Our study is the first to our knowledge that fully describes the presence of pain in community-dwelling manual wheelchair users with SCI, in a South African context. Pain has been reported according to the different types, locations and furthermore, the behaviour of pain was accompanied by a neuropathic pain questionnaire (DN4) to confidently conclude that indeed, neuropathic pain was the most common pain reported by the participants. To minimise selection bias, all potential participants from the four databases were contacted to invite them to participate in our study until sample size was reached. All findings from our study, irrespective of whether they are perceived as positive or negative, are reported to control publishing bias (Pannucci & Wilkins 2010). To add to the strength of our study, the sample was limited to people with paraplegia, in order to understand pain in a homogenous population. Nevertheless, generalisation of our results to people with tetraplegia should be done with caution.

### Implications and recommendations

Because of the fact that neuropathic pain being significantly associated with higher pain severity, and studies reporting pain severity to increase with more years living with SCI, it is prudent that pain management interventions focus on employing strategies to eradicate pain as much as possible. This includes conducting thorough assessments of pain and prescribing appropriate management interventions for the specific pain type complaints. Our study has confirmed what international and other local studies have reported that pain is common after SCI. Further research can be conducted to determine the interference of pain and the impact of pain on quality of life, community reintegration and functioning in a South African context.

## Conclusion

Pain after SCI is common and although several types can develop throughout the body at varying intensities, neuropathic pain burning in nature and in the legs below the level of injury is the most common pain in community-dwelling manual wheelchair users. Participants with complete SCI reported higher pain severity than those with incomplete SCI. Furthermore, neuropathic pain severity is reported as being higher than musculoskeletal pain severity.
